# Development of a Human Physiologically Based Pharmacokinetic (PBPK) Toolkit for Environmental Pollutants

**DOI:** 10.3390/ijms12117469

**Published:** 2011-10-31

**Authors:** Patricia Ruiz, Meredith Ray, Jeffrey Fisher, Moiz Mumtaz

**Affiliations:** 1 Computational Toxicology and Methods Development Laboratory, Division of Toxicology and Environmental Medicine, Agency for Toxic Substances and Disease Registry, Atlanta, GA 30333, USA; E-Mail: mgm4@cdc.gov; 2 Department of Epidemiology and Biostatistics, Arnold School of Public Health, University of South Carolina, Columbia, SC 29208, USA; E-Mail: mere2110@yahoo.com; 3 USFDA, National Center for Toxicological Research, Jefferson, AR 72079, USA; E-Mail: jeffrey.fisher@fda.hhs.gov

**Keywords:** volatile organic compounds, VOCs, metals, PBPK, toxicokinetic, National Health and Nutrition Examination Survey (NHANES)

## Abstract

Physiologically Based Pharmacokinetic (PBPK) models can be used to determine the internal dose and strengthen exposure assessment. Many PBPK models are available, but they are not easily accessible for field use. The Agency for Toxic Substances and Disease Registry (ATSDR) has conducted translational research to develop a human PBPK model toolkit by recoding published PBPK models. This toolkit, when fully developed, will provide a platform that consists of a series of priority PBPK models of environmental pollutants. Presented here is work on recoded PBPK models for volatile organic compounds (VOCs) and metals. Good agreement was generally obtained between the original and the recoded models. This toolkit will be available for ATSDR scientists and public health assessors to perform simulations of exposures from contaminated environmental media at sites of concern and to help interpret biomonitoring data. It can be used as screening tools that can provide useful information for the protection of the public.

## 1. Introduction

Default consumption values of air, water, soil, and foods are often used to estimate exposures to environmental pollutants from different routes of exposure. In addition, there is uncertainty regarding the amount of the chemical to which a person is exposed that is absorbed into the body and distributed to organs and tissues. Physiologically based pharmacokinetic (PBPK) models are being used to duplicate biological and physiological processes. These models may increase the accuracy of calculating the internal dose in tissues by use of such measures as blood or urine levels [1–3]. For these reasons, PBPK models can be used for extrapolating varied routes, doses, and species [4–7].

Even though multiple PBPK models are available they are too complex for field application by health risk assessors. An additional challenge is they are in multiple simulation languages for which advance education and training is required. Thus, translational research is needed to make such models accurate and accessible to workers in easy-to-use formats. The Agency for Toxic Substances and Disease Registry (ATSDR) has undertaken a project to convert and recode available, published PBPK models from multiple simulation languages into a single one that is easy to learn and operate. A library of models for certain ATSDR priority pollutants, such as volatile organic compounds (VOCs) and metals, has been developed, employing Berkeley Madonna software version 8.01 for Windows, Kagi Shareware, Berkeley, CA, USA for simulation and optimization because of its ease of application, economical multi-user license, and faster compilation properties [8]. This toolkit will assist researchers and risk assessors to assess potential chemical health effects. These models are not intended to be state-of-the-art models with metabolites or the latest version of a PBPK model. They should, however, be sufficiently vetted to allow health assessors to predict the consequences of complex exposures in terms of internal doses and their health implications. The models, including a basic training module, will be freely available on the Computational Toxicology and Methods Development Laboratory Web page upon the project’s completion. Specifically, ATSDR staff will be provided initial training in the advantages and limitations of the models available in the human PBPK toolkit. This article focuses on the project achievements to date, including the recoding of human PBPK/PK VOCs and metal models [9–11].

## 2. Methods

We first conducted a review of the literature to identify available human PBPK models for the chemicals of interest. The PBPK models varied in their complexity. They contained different numbers of compartments (e.g., liver, kidney, and other organs) and metabolites, and they were developed by use of different simulation languages, such as MatLab™, Simusolve, and AcslX™. Model selection was based in part on the number of data sets used to calibrate and evaluate the model, the model’s maturity (number of predecessor models from which the model was derived), and the experience of the authors. The models’ availability, performance, accuracy, and reproducibility also played a role [12–23]. Each model was constructed by use of flow-limited compartments describing the mass balance of the chemicals in multiple tissues. All compartments were described as well-mixed and flow-limited. All the recoded models, unless otherwise specified, allow simulation of different routes of exposure, either individually or simultaneously.

We derived a generic model that could be used for several VOCs, including benzene (BEN), carbon tetrachloride (CCl_4_), dichloromethane (DCM), perchloroethylene (PCE), trichloroethylene (TCE), and vinyl chloride (VC). This model was based on an individual model that met the study criteria mentioned above [18–23]. Only parent compound data sets and accompanying simulations were extracted from figures by use of Grab It! XP2 [24]. Original model simulations for metabolites and metabolite data were not included in this version of the PBPK VOCs toolkit development. Including metabolites is a critical future improvement for use of the PBPK models in dose-response assessments, when toxicity is mediated by metabolite formation.

For metals, arsenic, cadmium, and mercury models were recorded as original published. Because of chemical-specific kinetics differences in each model, no attempt was made to develop a generic model [12–17].

### 2.1. Model Structure and Physiological Parameters

We constructed a seven-compartment generic VOCs model with blood, fat, skin, kidney, liver, rapidly and slowly perfused tissue compartments, plus a gas exchange compartment. Elimination and absorption were accounted for by incorporating a gas exchange and a skin compartment accounting for portal of entry and loss of the VOC from the body; the liver for metabolism, including first pass metabolism after oral intake; the fat as a reservoir; and the kidney as a possible target organ and potential excretory organ. Distribution to the remaining tissues was grouped on the basis of rates of blood perfusion, to maintain mass balance. All compartments were described as well-mixed and flow-limited.

Human physiological parameters used in this study such as tissue volumes, alveolar ventilation, rate of metabolism, cardiac output and chemical specific parameters were taken from the literature [18–36]. Only TCE [30] and PCE [36] skin:blood partition coefficients have been reported. Thus for the other VOCs, the TCE skin:blood partition coefficient values were used. To describe dermal uptake of other VOCs dissolved in water, we would need estimates of skin:water partition coefficients and Kp (permeability constant) values. The dermal exposure route is available in the model, but lack of chemical-specific parameters and human pharmacokinetic data for this route of exposure is a major challenge for many of the VOCs. Several approaches, such as Quantitative Structure Activity Relationship (QSAR), have been used to fill this data gap and could be employed with these models to estimate the dermal contribution to exposure. In the current model version we also did not include original-model simulations for metabolites and metabolite data. Nevertheless, a critical future improvement for this model’s post-screening use is incorporation of metabolite information, particularly when toxicity is mediated by metabolite (s).

The methylmercury model was patterned after Carrier *et al*., 2001. This model consists of blood, liver, kidney, brain, hair, urine, and feces compartments [13,14]. The arsenic model was patterned after El-Masri and Kenyon [12]; this model consists of interconnected sub-models for inorganic arsenic III and V and its metabolites, monomethyl arsenic (MMA) and dimethylarsenic (DMA). It includes compartments for the lung, liver, GI tract, kidney, muscle, brain, skin, and heart [24]. The cadmium model was based on the initial work of Nordberg-Kjellstrom, later modified by Choudhury *et al*., 2001 and Diamond *et al*., 2003 [15,16].

The cadmium toxicokinetics that used differential equations were to describe the inter-compartmental transfers of cadmium, and the growth algorithms for males and females and corresponding organ weights were used to calculate age-specific cadmium concentrations from tissue cadmium burdens.

Human physiological and chemical-specific parameters of As, Hg, and Cd were taken from the original published model [12–17,27–31]. These models can simulate two routes of exposure, inhalation and oral, either individually or simultaneously.

### 2.2. Model Evaluation

Assessment of our generic VOCs PBPK model was first performed by comparison of the published human kinetic data for each VOC and our recoded version of the published model. To further insure the reliability of our generic VOCs model, the area under the concentration curve (*AUC*) for blood or exhaled breath was calculated for each VOC, using both our generic VOCs model and the original model. Predicted *AUC* values in blood or breath for each VOC were then compared to the data-derived *AUC* values (using the trapezoidal rule) in blood or breath. For each VOC, the fit was expressed as a ratio (*AUC*_r_) that equaled the *AUC* value for the published model or for our generic VOCs model divided by the *AUC* value computed from the kinetic data. We recognize shortcomings in using data-derived *AUC* values, in that they may either inflate or deflate the probable *AUC* values, depending on the quality of the data. Nevertheless, our interpretation of the *AUC* ratios was that the closer the value was to one, the better the agreement between measured and model prediction.

For each kinetic time course dataset, we also calculated the mean of the sum of the squared differences (MSSDs) between model prediction and observation. We computed MSSD by squaring the difference between a measured data point and the value of the simulation at the corresponding time. We summed these squares and then divided the sum by the number of data points. The MSSD was thus determined for both the published model and for our generic VOCs model. One interpretation is that the lower the MSSD value, the better the fit. However, the absolute values of the data can skew the results; thus, professional judgment is considered important in deciding the quality of the fits between model prediction and observation.

Assessment of the PBPK metals models was conducted by comparison of human data sets to recoded and published model simulations. We achieved the assessment by calculating a value for percent median absolute performance error (MAPE%) on the basis of estimates of performance error (PE) [14]. The accuracy of the prediction was measured by root median-square performance error (RMSPE%) as:

$$\text{RMSPE}\%=\sqrt{\sum_{i=1}^{n}\frac{{\text{PE}}^{2}}{n}}$$

where *n* is the total number of data points. We also calculated the correlation coefficient between *C*_measured_ and *C*_predicted_.

The robustness of each of the recoded models was also studied by use of the sensitivity ratio (SR) approach. This type of sensitivity analysis shows the strength and relevance of the inputs in determining the variation in the output. The SR ratios for each input–output pair of variables were calculated [37]. A positive sensitivity ratio indicates that an increase in the input value results in an increase in the output value. A negative sensitivity ratio indicates the opposite effect.

## 3. Model Applications

The Centers for Disease Control and Prevention’s (CDC) National Health and Nutrition Examination Survey (NHANES) provides a representative sample of environmental testing on blood and urine specimens. With the NHANES data, CDC’s Environmental Health Laboratory conducts biomonitoring for over 200 chemicals [38]. All the VOCs as well as metals for which we have recoded models in our human PBPK toolkit have been reported in the Fourth National Report on Human Exposure to Environmental Chemicals [38].

We used our VOCs PBPK model to simulate various Minimal Risk Levels (MRLs) exposures for each of the VOCs for which biomonitoring data on human blood levels were available from the Fourth National Report on Human Exposure to Environmental Chemicals [38]. MRLs are an ATSDR estimate of daily human exposure to a hazardous substance at or below which that substance is unlikely to pose a measurable risk of harmful (adverse), noncancerous effects [39,40]. MRLs are calculated for an exposure route (inhalation or oral) over a specified period (acute, intermediate, or chronic). MRLs simulations were run as a combination of continuous 24 h inhalation and oral ingestion exposures (equally spaced four times a day) at the MRLs for acute (14 days), intermediate (365 days), and chronic (>365 days) durations. Steady-state VOC concentrations in venous blood were then compared to NHANES data by use of these simplified assumptions about exposure frequency and duration. If the measured NHANES blood levels are below those estimated from the simulations, the exposures are regarded as safe.

For the metals models, our toxicokinetic recoded model for cadmium was used to interpret the Cd urinary concentrations reported in the Fourth National Report on Human Exposure to Environmental Chemicals. Oral ingestion exposures were simulated by use of the geometric mean dietary Cd intakes for each of the sex-age stratified datasets [9].

## 4. Results and Discussion

**Figure 1:**
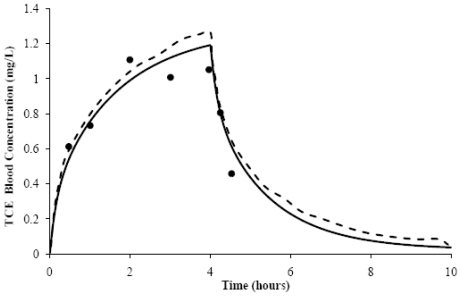
Trichloroethylene (TCE) blood concentrations (●) measured over time, following a 4 h, 50 ppm TCE inhalation exposure (Fisher *et al*. 1998 [22]). The original simulation (---) and our generic VOCs model simulation (—) are also shown.

The seven-compartment generic VOCs model we constructed adequately reproduced simulations for all the VOCs. The simulations included various exposure scenarios for multiple routes and varying times of exposure for exhaled breath and arterial blood concentrations, as determined by the *AUC* ratios (*AUC*_r_) and the MSSD values (Table 1). A general acceptability within the modeling community is that the closer the value of the *AUC*_r_ to one, the better the agreement between measured and model predictions. Similarly, the lower the MSSD value, the better the fit between the absorption, elimination, and steady state curves, as illustrated by the simulations shown for TCE (Figure 1). This figure shows the original model’s and our generic VOCs model’s comparative simulations of predicted arterial blood concentration following a 4-h, 50-ppm TCE inhalation exposure. These results show that our generic VOCs model simulation of TCE inhalation exposure in male humans compared favorably to the original model [22]. For both models, the *AUC*_r_ values were 0.8 (Table 1); the MSSD values were similar and low—0.0089 for the original model and 0.0095 for our generic VOCs model. As seen in Table 1, sometimes the models under- or over-predicted, but these differences are within the parameters of variability common for exposure assessment in risk assessments.

**Figure 2:**
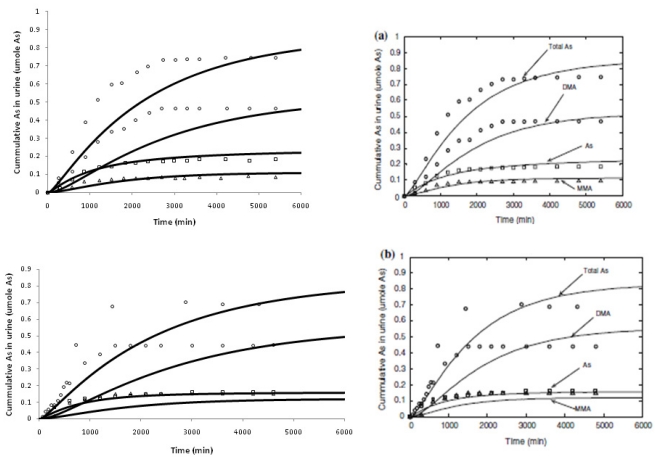
Total As, monomethyl arsenic (MMA), and dimethylarsenic (DMA) cumulative urinary excretion in human volunteers exposed to 100 μg As in the form of sodium arsenate (panel **a**) and sodium arsenite (panel **b**). Our recoded model simulation (Left, solid line) *versus* the reworked original simulation by El-Masri and Kenyon, 2008 [12] (Right, solid line).

All the recoded metal models adequately simulated experimental human data found in the published literature [12–17]. As shown in Figure 2, the arsenic model predicted cumulative urinary excretion of total As and its two methylated metabolites in humans adequately, and the arsenic model is in good agreement with the original model. Performance evaluation was measured using MAPE%, MPE%, and RMSPE% for the three metals [10]. As is true with every PBPK and toxicokinetic model, these models try to capture various biological processes of absorption, distribution, metabolism and excretion (ADME) on the basis of available experimental data. Such simplifications sometimes can lead to shortcomings. Thus, model assumptions should be clearly understood and simulation interpretation should be put in perspective, with actual toxicity findings or reported facts.

After evaluation of each recoded model, we tried to interpret the findings of the report by using these models as a screening tool. The generic VOC PBPK model was used to estimate the blood concentrations for the available MRL values of each of the specific VOCs (Table 2), and the toxicokinetic Cd model was used to predict the urinary cadmium concentrations for non-smokers in the U.S. populations (Table 3). These results illustrate that the human PBPK toolkit can be used as an initial screening tool for some of the most prominent VOCs, and it can help direct further detailed analysis when such analysis is warranted. The application of the cadmium model demonstrated that it too can be used to predict urinary excretion on the basis of cadmium intake across various age groups, except for the elderly (>60 years of age), where absorption from the gut is a known compounding factor, as shown by other published models [15,16].

In this paper, we have reviewed the progress that has been made at ATSDR to make available PBPK models by bridging the gap between model development and use. We did this through harmonizing efforts of recoding the best available published PBPK models from multiple simulation languages into a single simple simulation language—Berkeley Madonna—that when completed will be packaged into a human PBPK toolkit. Currently, the recoded models include three high-ranking metals and some commonly encountered VOCs from ATSDR’s priority list of environmental contaminants. We have demonstrated that the toolkit can be used in the assessment of biomonitoring results as a screening tool. The human PBPK toolkit that is being developed at ATSDR has the major advantage that it can be applied in the field by practitioners of risk and health assessments.

The use and acceptance of computational tools such as the human PBPK toolkit in the decision-making process should be acquired through an interaction between the model developers and model users. These interactions will lead to an increased application of such tools in the field and an increased awareness of their advantages and limitations. Such interactions and awareness will promote the integration of the toolkit into the alternative tools available for decision-makers. Their optimal use can only be realized through information exchange and shared expertise. The only way to promote their use is to make such tools easy to use and apply. We have shown that models available in multiple, simulation languages can be recoded into one simulation language. Thus, the end-user has to learn only one simple language, rather than a multitude of computer languages, to derive the predictions needed for risk assessments. These types of efforts will allow validation and verification of results to give the user confidence of their integration in the overall risk assessment processes.

In conclusion, computational toxicology is a growing field that will produce new and innovative tools that will become increasingly available for chemical risk assessment. High throughput screening and *in vitro* testing are drastically changing the testing strategies in toxicology. They are at the threshold of creating the next generation of computational tools. Before the nextGen tools become available, it is imperative that the risk assessors start using PBPK modeling and similar computational tools that have been developed during the past few decades. To keep pace with the changing science, we should continue to develop libraries of predictive tools that will help in the hazard identification and risk assessment process; otherwise, there is a real concern that the tools that are being employed might not keep pace with advances in science or recommendations of such organizations as the National Academies of Science (NAS). These organizations are recommending a totally new approach, such as pathway analysis, and systems approaches to evaluate the consequences of exposure to chemicals.

## Figures and Tables

**Table 1:** Physiologically Based Pharmacokinetic (PBPK) volatile organic compounds (VOCs) Model Comparison.

	*AUC*_r_		MSSD	

VOCs	Generic Model	Original Model	Generic Model	Original Model
**BEN**^a^	0.9	1.6	0.0008	0.0009
**CCl_4_**^b^	2.5	1.9	0.4515	0.2344
**DCM**^c^	1.1	1.1	3.8214	1.1722
**PCE**^c^	0.6	0.8	0.0805	0.0164
**TCE**^c^	0.8	0.8	0.0095	0.0089
**VC**^b^	1.2	1.1	0.1875	0.1831

BEN, benzene; CCl_4_, carbon tetrachloride; DCM, dichloromethane; PCE, perchloroethylene; TCE, trichloroethylene; VC, vinyl chloride.

a μM;

b ppm;

c mg/L.

**Table 2:** Comparison of Minimal Risk Level (MRL) simulated blood concentration of each solvent, assuming simultaneous inhalation (24 h/day) and oral ingestion (4 drinking bouts per day) to the measured blood concentration of solvent reported by National Health and Nutrition Examination Survey (NHANES) 2003–2004. The simulated solvent exposure is set to the MRL for inhalation of the solvent in air and ingestion of the solvent in water.

	BEN^+^	CCl_4_^+^	DCM^+^		PCE^+^	TCE^+^	VC^+^
MRL*	0.003**/**0.0005	0.03**/**0.007	0.6**/**0.2	0.3**/**0.06	0.2**/**0.05	2**/**0.2	none
Exposure Duration	Chronic	Intermediate	Acute	Chronic	Acute	Acute	----
**PBPK MODEL**	**Blood Concentration (ng/mL)**						
Predicted Peak	0.04	0.40	18.12	6.70	10.76	111.65	----
NHANES**	**Blood Concentration (ng/mL)**						
	0.260 (0.210–0.320)	<LOD	<LOD		0.140 (0.091–0.300)	<LOD	ND **
Limit of Detection (LOD)	0.024	0.005	0.07		0.048	0.012	ND

Ben^+^, benzene; CCl_4_ ^+^, carbon tetrachloride; DCM^+^, dichloromethane; PCE^+^, perchloroethylene; TCE^+^, trichloroethylene; VC^+^, vinyl chloride;

* Inhalation concentration (ppm)**/**Oral ingestion rate (mg/kg-day);

** NHANES 2003–2004. 95th percentiles of blood concentration (in ng/mL) for US population, ND = Not Done.

**Table 3:** Dietary cadmium intake, model predictions, and geometric mean urinary cadmium concentrations in nonsmoking male U.S. population (National Health and Nutrition Examination Survey: NHANES 2003–2004).

Age group (years)	Males			Females		
	* Urinary Cd (μg/g creatinine)		Cd Intake GM (μg/day)	* Urinary Cd (μg/g creatinine)		Cd Intake GM (μg/day)
	Measured	Predicted		Measured	Predicted	
6–11	0.088 (0.071−0.11)	0.101 (0.071−0.11)	15.0	0.088 (0.072−0.108)	0.172 (0.152−0.188)	13.5
12–19	0.074 (0.066−0.083)	0.087 (0.078−0.095)	19.7	0.103 (0.089−0.118)	0.163 (0.136−0.190)	15.1
20–39	0.125 (0.114−0.137)	0.137 (0.082−0.190)	22.4	0.179 (0.159−0.202)	0.285 (0.182−0.386)	16.2
40–59	0.208 (0.184−0.234)	0.214 (0.188−0.241)	22.1	0.342 (0.305−0.383)	0.427 (0.377−0.477)	16.5
≥60	0.366 (0.324−0.414)	0.226 (0.221−0.232)	17.6	0.507 (0.460−0.558)	0.453 (0.447−0.459)	14.4

* From Choudhury *et al*., 2001 [15]. GM = geometric mean.
